# Micronized, Microencapsulated Ferric Iron Supplementation in the Form of >Your< Iron Syrup Improves Hemoglobin and Ferritin Levels in Iron-Deficient Children: Double-Blind, Randomized Clinical Study of Efficacy and Safety

**DOI:** 10.3390/nu13041087

**Published:** 2021-03-26

**Authors:** Aida Zečkanović, Marko Kavčič, Tomaž Prelog, Alenka Šmid, Janez Jazbec

**Affiliations:** 1 Department of Pediatric Hematology and Oncology, University Children’s Hospital, University Medical Centre Ljubljana, Bohoričeva ulica 20, 1000 Ljubljana, Slovenia; aida.zeckanovic@kclj.si (A.Z.); marko.kavcic@kclj.si (M.K.); tomaz.prelog@kclj.si (T.P.); 2 The Chair of Clinical Biochemistry, Faculty of Pharmacy, University of Ljubljana, Aškerčeva cesta 7, 1000 Ljubljana, Slovenia; alenka.smid@ffa.uni-lj.si

**Keywords:** iron deficiency, pediatric, children, supplementation, microencapsulated iron, syrup, liquid, food supplement

## Abstract

A major problem of oral iron supplementation efficacy in children is its tolerability and compliance. We aimed to determine the safety and efficacy of a novel food supplement >Your< Iron Syrup in the replenishment of iron stores and improvement of hematological parameters in iron-deficient children aged nine months to six years. We randomized 94 healthy children with iron deficiency in a ratio of 3:1 to either receive >Your< Iron Syrup or placebo. A 12-week supplementation with >Your< Iron Syrup resulted in a significant increase in ferritin and hemoglobin levels as compared to placebo (*p* = 0.04 and *p* = 0.02). Adverse events were reported with similar frequencies across both study arms. >Your< Iron Syrup represents an effective, well-tolerated, and safe option for the management of nutritional iron deficiency in children.

## 1. Introduction

Iron is an essential micronutrient indispensable for numerous metabolic processes. Depletion of iron from body stores manifests as decreased levels of serum ferritin and is regarded as iron deficiency (ID). During ID, clinical symptoms might be absent or minimal. If not reversed, ID progresses to iron deficiency anemia (IDA) presenting with low hemoglobin concentration and microcytosis. At this stage, pallor, irritability, fatigue, lethargy, and pica become evident [1,2]. Since iron also plays a role in essential neurologic processes such as neurotransmitter synthesis and myelination, ID, with or without anemia, is thought to impact cognitive, behavioral, and motor development. While correction of iron status has the potential to improve these outcomes, some impairment may be irreversible [3]. Therefore, it is substantial to ensure adequate iron intake and manage ID when identified [4].

ID is the most common nutritional problem worldwide, mainly because of malnutrition in developing countries. In developed countries, significant proportions of ID and IDA are attributable to eating habits, such as vegan or vegetarian diets or scarce intake of red meat. Regardless of eating habits, ID also prominently arises in individuals with increased physiological requirements for iron, such as infants and young children [2,5,6].

Healthy, full-term, normal birth weight infants are endowed with a sufficient amount of iron to cover the needs for the first 4 to 6 months of their life independent of dietary intake. After this, infants become critically dependent on dietary iron, and because of their rapid growth, iron requirements are higher than during any other period of life [4]. Infants under 2 years of age are most vulnerable to the development of ID, and collectively, children below 5 years bear the largest burden of IDA [6].

To ensure adequate intakes of iron, successfully manage ID, and prevent its deterioration towards IDA, dietary interventions can be combined with oral iron supplementation. However, iron-containing preparations in the form of syrups or drops have an unpleasant taste, may stain the teeth, and cause gastrointestinal disturbances, all of which decrease compliance to the intervention and thus limit its effectiveness [7]. Therefore, new products with increased palatability and improved side-effect profile are constantly being sought for.

In the present study, we sought to evaluate safety and efficacy of a novel iron-containing food supplement, >Your< Iron Syrup, developed especially for children, to be used in the management of ID.

## 2. Materials and Methods

### 2.1. Study Design

This was a double-blind, randomized, placebo-controlled study of efficacy and safety of a novel liquid iron dietary supplement >Your< Iron Syrup in children. The study was carried out at 15 Slovenian pediatric primary care units between December 2017 and June 2020. The study was conducted following the International Conference on Harmonization Good Clinical Practice guidelines, the Declaration of Helsinki as amended, as well as all current Slovenian and European guidelines and regulations for clinical research. The study protocol and relevant documents were approved by the National Medical Ethics Committee of the Republic of Slovenia (number 0120-132/2017) and by internal ethics boards of respective research centers (where required) before the initiation of the study. This study was registered at www.ClinicalTrials.gov #NCT04713943.

### 2.2. Study Population

**Figure 1:**
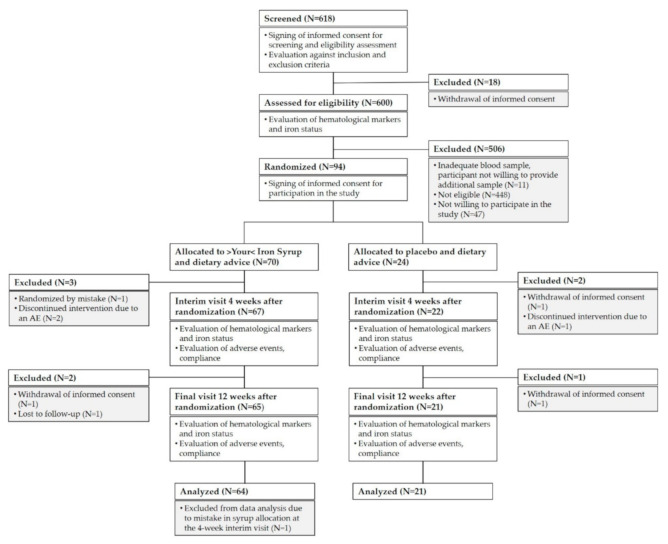
Flow diagram of the participant’s progress through the study.

The study was initiated at 17 research centers, screening of children was carried out at 15 of these centers, and 10 centers succeeded in enrolling any children into the study. A total of 618 healthy children aged nine months to six years were screened for eligibility during regular health check-up visits. Of these, 94 children were enrolled in the final cohort. Most of the screened children were not eligible for the study because they did not meet the laboratory inclusion criteria (see Figure 1). We included only children with iron deficiency presenting either without anemia or with mild microcytic hypochromic anemia not requiring medication (as assessed by ferritin level ≤20 µg/L and hemoglobin (Hb) ≥ 100 g/L in a capillary blood sample). We excluded any child with anemia that was not due to iron deficiency, any child with known allergies to any of the ingredients of >Your< Iron Syrup, any child with chronic disease and any child with a medical, mental, or physical state that, in the opinion of the investigator, would be incompatible with taking the investigational product or with the course of the study. Vegans and those already taking iron-containing dietary supplements or medications were also excluded from participation, as well as those taking part in another clinical study at screening or less than one month before screening.

### 2.3. Study Product

Study participants were randomly assigned to receive either >Your< Iron Syrup or placebo. Both products were developed, manufactured, and supplied by PharmaLinea Ltd. (Ljubljana, Slovenia) under the ISO 22000 standard and tested by independent external accredited laboratories for the content of heavy metals, active ingredients, and microbiological integrity. Products were matched for appearance, smell, taste, and packaging and sets of bottles as well as their respective outer packaging were coded by a unique two-letter-one-number code. A unique syrup code was assigned to each study participant throughout the study. >Your< Iron Syrup contained 14 mg of elemental iron in the form of branded micronized, microencapsulated ferric iron (Qfer), 0.7 mg of vitamin B6, and 1.25 µg of vitamin B12 as active ingredients per 5 mL of the product. Placebo syrup did not contain any iron but was otherwise exactly matched in composition to >Your< Iron Syrup.

### 2.4. Study Procedures

Screening of children for assessment of their eligibility for participation in the study proceeded at regularly scheduled health check-up visits at children’s pediatricians. Children were assessed against inclusion and exclusion criteria and 0.5 mL of capillary blood was sampled for determination of complete blood count (CBC) and plasma ferritin, both being markers of iron status. Since ferritin concentration can be elevated in case of inflammation, C-reactive protein (CRP) concentration was determined to exclude individuals with high ferritin due to coexistent inflammation. Ineligible children were excluded from further participation in the study and eligible children were invited for an enrolment visit. Separate free and voluntary written consents for screening as well as for enrolment of children in the study were obtained from their parents before any screening- or study-related assessments and/or procedures were performed.

At enrolment visit, children were randomized to either >Your< Iron Syrup or placebo arm in a 3:1 ratio. The randomization was centralized and performed by an independent study monitor who assigned each child with a unique randomization number that was also used on the syrup packaging. Both investigators and parents were blinded to the allocated intervention for the whole duration of the study. At enrolment, each child’s weight was recorded to determine the required amount of syrup (1 mg of elemental iron per kg of body weight) to be dispensed to last until the next scheduled study visit. The dose for each child was rounded to the nearest 1 mL unit. At follow up visit, the dose of syrup was modified in response to weight change. All parents also received counseling on their child’s age-appropriate iron-rich diet and were provided a paper-based study diary to enter any modifications implemented to their child’s diet, any adverse events (AE) and missed doses of syrup. Enrolment visit was followed by an interim follow-up visit 4 weeks (±3 days) later and a final follow-up visit after a period of 12-weeks (±3 days) of supplementation. At interim follow-up visit, all study products, dispensed at the previous study visit, were collected from the subjects, and compliance to study interventions was calculated. Investigators checked returned study diaries for any AEs and assessed the tolerability of supplementation. A new set of study supplements was issued to each child, based on their newly measured weight, to last until the final follow-up visit, as well as a new study diary. A sample of capillary blood was drawn at interim and final follow-up from each participant to monitor the efficacy of intervention through changes in CBC and plasma ferritin (in conjunction with CRP) parameters. All blood samples were analyzed at a central accredited study laboratory (Adria lab d.o.o., Ljubljana, Slovenia). A paper-based case report form was used to capture information recorded in the source data of each study participant for each study visit. Subject confidentiality was ensured throughout the study by assignment of a unique study number to each participant screened or enrolled in the study.

### 2.5. Study Outcomes

The primary outcome was the assessment of the efficacy of >Your< Iron Syrup in the alleviation of iron deficiency, defined as a share of children having ferritin value >20 µg/L after 12 weeks of supplementation. As secondary outcomes, a share of children having ferritin value >20 µg/L after 4 weeks of supplementation was analyzed and average changes in ferritin (coupled to measurements of CRP), hemoglobin, mean corpuscular volume (MCV), mean corpuscular hemoglobin (MCH), and mean corpuscular hemoglobin concentration (MCHC) after 4 and 12 weeks of supplementation were recorded. Incidence, severity, and types of AEs reported during the 12-week intervention period were assessed as safety outcomes. The severity was defined as mild (noticeable, but easily manageable), moderate (presents a minor hindrance in everyday life), and severe (severely hinders a child’s ability to carry out daily tasks). The participating researchers graded each AE as either not linked, possibly, probably, or definitely linked to the supplement.

### 2.6. Sample Size and Statistical Analysis

The sample size was calculated through a sample size equation that projected the study to have 80% statistical power and accounted for a 15% drop-out rate and 5% type I error. We took into account the 3:1 randomization ratio and assumed that after 12 weeks of supplementation ferritin levels would be >20 µg/L in 50% of the participants in the placebo arm and 85% of the participants in the >Your< Iron Syrup arm. A total of 92 participants were determined to be required (69 in >Your< Iron Syrup arm and 23 in the placebo arm). SPSS Statistics software, version 22 (IBM Corp., Armonk, NY, USA), was used for the statistical analysis. We used an independent-samples student’s *t*-test to compare the hematological characteristics between the two groups. We used a paired samples t-test for comparison of baseline values to end-point values of hematological parameters. To compare the side effect scores, we used either the Chi-square test or Fisher’s exact test. The level of significance was set at 0.05.

## 3. Results

### 3.1. Study Population

A total of 618 participants signed informed consent for screening. Of these, 600 were assessed for eligibility. A total of 141 children met eligibility criteria, and 94 were enrolled in the study (47 were not willing to participate in the study). Of the 70 children who were randomized to >Your< Iron Syrup arm, 64 children (91%) completed the study, and 3 children were withdrawn prior to interim follow-up due to randomization mistake (one child) or due to an AE (two children). Another two children withdrew between interim and final follow-up (parents of one child withdrew informed consent and one child was lost to follow-up). One child was excluded from analyses because of a mistake in syrup allocation at the interim follow-up visit. In the placebo arm, 21 out of 24 individuals (88%) completed the study (parents of two children withdrew informed consent and one child discontinued the intervention due to an AE). For AE analysis, we excluded the children that withdrew their informed consent and were lost to follow-up but included the children who withdrew from the study because of an AE (two children in the >Your< Iron Syrup arm and one in the placebo arm). Figure 1 summarizes group allocation and progression through the study.

Participant demographics are shown in Table 1. There were no significant differences in the baseline demographic characteristics between the study arms.

### 3.2. Baseline and Follow-Up Hematological Characteristics and Iron Status

Table 2 shows baseline iron status indices as well as values after 4 and 12 weeks of intervention. After 12 weeks of supplementation, ferritin levels were significantly higher in both arms compared to baseline values (>Your< Iron Syrup *p* = 0.00 and placebo *p* = 0.01), and 55% (35/64) of children in the >Your< Iron Syrup arm had end-point ferritin levels above 20 µg/L, compared to 43% (9/21) of the children in the placebo arm. When compared with the Chi-square test, the difference between groups was not statistically significant (*p* = 0.35).

The endpoint mean ferritin (*p* = 0.04, unequal variances assumed), hemoglobin level (*p* = 0.02), hematocrit (*p* = 0.02), and erythrocyte counts (*p* = 0.03) were significantly higher in >Your< Iron Syrup compared to the placebo arm. Erythrocyte counts were already significantly higher in the >Your< Iron Syrup group after four weeks of supplementation. The differences between MCV, MCHC, and MCH were not statistically significant after 4 or 12 weeks.

### 3.3. Adverse Events

The most frequent adverse events were common pediatric infectious diseases and gastrointestinal disturbances (constipation, darker stool, diarrhea, abdominal pain, and vomiting) (Table 3).

In the >Your< Iron Syrup arm, 63 children reported 172 AEs; 83% were mild, and 88% were assessed as unrelated to the supplementation. One severe AE (abdominal pain) in the >Your< Iron Syrup arm was evaluated as possibly linked to the supplementation. Eleven mild and five moderate gastrointestinal disturbances were assessed as either possibly or probably linked to >Your< Iron Syrup. Another three mild AEs (skin rash, teeth discoloration, and cough) were evaluated as possibly linked to the supplementation.

In the placebo arm, 19 children reported 42 AEs, 76% of which were mild and 76% were assessed as unrelated to the supplementation by the participating pediatricians. Seven mild and three moderate gastrointestinal AEs were evaluated as possibly (*n* = 9) or probably (*n* = 1) linked to the supplementation. There were no severe AEs reported.

Gastrointestinal AEs were the most frequently evaluated as possibly or probably linked to the supplementation in children in both >Your< Iron Syrup and placebo arms. The differences between the two arms were statistically significant, with a higher proportion of supposedly related AEs in the placebo arm (56% vs. 21%, *p* = 0.03).

There was no significant difference in the frequency of children that experienced AEs between the study arms (*p* = 0.16) and no significant differences in the frequency, type of AE (*p* = 0.64) and distribution of mild, moderate, and severe AEs (*p* = 0.07).

None of the infectious AEs were deemed to be related to the study intervention.

Overall, two subjects in the >Your< Iron Syrup arm and one subject in the placebo arm decided to prematurely exit the study due to AEs. All three AEs were moderate gastrointestinal disturbances. The researchers evaluated all three as probably connected to the supplementation.

During the study, two serious AEs were reported in two test subjects, randomized to >Your< Iron Syrup arm. Both were gastrointestinal (abdominal pain and gastroenteritis), and one was deemed by the researchers as unrelated and the other as possibly related to the supplement. Both children completed the study despite these AEs.

### 3.4. Compliance

The mean compliance in both arms was 92% according to recorded missed doses of syrup in the study diaries. There were no statistically significant differences in reported compliances between the two study arms (*p* = 0.85) compared by an independent samples *t*-test.

Compliance to >Your< Iron Syrup and placebo, calculated from volumes in returned syrup bottles, is given in Table 4. Of the participants, 80% were 100% compliant and 97% were at least 80% compliant with the intervention, without significant differences between the arms.

>According to the analysis of study diaries, 4 (6.3%) of children in Your Iron Syrup arm and zero of children in the placebo arm had modifications to increase iron intake introduced into their diet for the whole duration of the study. A total of 19 children (29%) in “Your Iron Syrup” arm and in 1 child (4.8%) in the placebo arm followed the suggested dietary advice 50% of the time. The differences between the two study arms are not significant for 100% compliance, but 50% compliance is statistically significantly lower in the placebo arm (Fisher’s exact test, *p* = 0.02). 

## 4. Discussion

Serum ferritin level is the most sensitive and specific test used for the identification of ID in the absence of inflammation or chronic disease [5]. The WHO sets ferritin threshold at <12 µg/L for children younger than 5 years and at <15 µg/L for 5- to 12-year-olds [8]. By picking higher cutoffs (ferritin level ≤20 µg/L), we gain higher sensitivity but loose on specificity. To limit the inclusion of false ID, CRP level was measured at enrolment visit, interim and a final follow-up to identify cases of higher ferritin due to concomitant inflammation. Throughout the study, there were 18 instances where the CRP was above 5 mg/L, with a maximum value of 22 mg/L. Their distribution did not significantly differ between the study arms.

In our study, only nine enrolled children presented with mild anemia as defined by a Hb level of less than 110 g/L (below 5 years of age) or 115 g/L (above 5 years of age) [9]. Most of the population thus consisted of children with either depletion of iron stores or iron-deficient erythropoiesis without anemia. Not having measured any of the early indicators of ID states, such as transferrin saturation, soluble transferrin receptor level, reticulocyte Hb, etc., we were unable to characterize our ID population in more detail. This must be acknowledged as a limitation of our study.

The results of the study indicate that it is feasible to correct iron status in an at-risk pediatric population by daily consumption of >Your< Iron Syrup. After 12 weeks of supplementation, ferritin level increased beyond 20 µg/L in 55% of children in the >Your< Iron Syrup arm. On the other hand, 57% of children receiving placebo retained suboptimal iron stores. Furthermore, ferritin levels after 12 weeks of supplementation improved significantly in >Your< Iron Syrup arm as compared to the baseline value as well as to placebo (*p* = 0.04). Moreover, Hb level, hematocrit, and erythrocyte count improved significantly as compared to placebo, and the difference in erythrocyte counts was already detectable after four weeks. There were, however, no changes in MCV, MCH, or MCHC in either arm. Although characteristic, these erythrocyte indices are derived from the entire circulating red cell mass and are relatively late indicators of erythropoiesis that substantially change only during IDA [10]. 

Generally, ID may proceed to IDA and persist without progression, or it may resolve spontaneously [5,11]. A study by Ullrich et al. demonstrated that only 4% of children diagnosed with ID progressed to IDA in 3 months following the diagnosis [12]. No data were presented on the share of the children who remained iron deficient or who had their ID reversed to an iron-replete state. Furthermore, reticulocyte hemoglobin and not ferritin was used as a screening and assessment tool for ID, and the population studied were 9- to 12-month-old infants, all of which prevents these results to be directly comparable to the results of our study. Based on ferritin and hemoglobin measurements, during our study, 14% of the children (three children) in the placebo arm progressed toward anemia (Hb below 110 g/L), 57% remained iron deficient, and in 43% of children ID spontaneously resolved. This is in contrast to >Your< Iron Syrup arm, where 55% of participants had sufficient iron stores and 4.7% (three children) progressed toward anemia (Hb below 110 g/L).

While 45% of the children in >Your< Iron Syrup arm remained iron deficient (ferritin value below or equal to 20 µg/L) even after the intervention had been completed, this may be explained by the relatively low dose of supplementation used in the study. Hb value may normalize on a small daily dose of iron, but this low dose seldom suffices for metabolic needs or the replenishment of iron stores in the body [13]. Similar studies of iron supplementation in children have reported higher efficacies within a 3-month supplementation period; however, much higher doses of iron (ranging from 2 mg/kg of iron per body weight per day to 6 mg/kg of iron per body weight per day) than the one applied in our study have been used [14,15,16]. Of particular importance, these studies were all performed in anemic subjects, whereas our study included children with ID without anemia. It is well established that iron absorption is negatively regulated by the regulatory hormone hepcidin, the level of which tends to be more profoundly reduced in IDA than in ID [17,18], thus possibly differentially affecting the degree of iron absorption in these instances. Significant effects of oral iron supplementation might therefore be evident at earlier time points in IDA as opposed to ID.

Our study results demonstrate that supplementation with Your Iron Syrup is effective in preventing the development of anemia in children with latent sideropenia and even shows a trend towards replenishing the iron stores. However, due to the small size of our sample, a larger study would be needed for a more definitive assessment.

Oral iron supplementation is almost always accompanied by side effects, often leading to poor compliance and consequently to reduced efficacy of the intervention. It is well known that after the initial recovery of Hb levels, an additional 3 to 6 months of supplementation are required for the repletion of iron stores and the normalization of ferritin levels [5]. Therefore, long-term compliance can only be expected when both side effects of daily supplementation are minimal and palatability is acceptable.

The most-commonly encountered adverse effects of oral iron supplementation are of gastrointestinal nature, including nausea, vomiting, abdominal pain, constipation, and diarrhea. Teeth discoloration and skin reactions may also occur [13]. In an attempt to minimize these adverse effects, >Your< Iron Syrup has been formulated to contain micronized encapsulated iron. Encapsulation of iron improves its overall tolerability, partly due to the ability to mask the unpleasant taste of iron, and partly due to the ability to reduce interactions of non-absorbed iron with the gastrointestinal mucosa [19] and possibly gut microbiota [20].

Parents of the children, included in our study, reported 214 adverse events during the 12-week study period, 172 in the >Your< Iron Syrup arm, and 42 in the placebo arm. The majority of these events comprised of gastrointestinal disturbances and concomitant infections. These events were reported with similar frequencies across both study arms and reflect the expected consequences of typical mild childhood illnesses in a healthy pediatric population with regular access to primary care. The majority of adverse events were mild, and their severity was comparable across both study arms. Gastrointestinal adverse events were most-commonly assessed by the researchers as being linked to the supplementation; however, interestingly, more so in the placebo group. This may be due to a high overall incidence of gastrointestinal disturbances in infants and small children, which encumbers the evaluation of the link between the event and the supplementation. Importantly, rare adverse events lend support to >Your< Iron Syrup as a safe option to intervene in children at risk for iron deficiency.

Dietary counseling is the first step in the management of mild ID. Although important, it may not be the most effective management strategy for ID in the general public. While the highly motivated individual may be able to embark on and sustain a rigorous dietary regimen to improve their iron status, supplementation is likely a more practical option for most. This holds especially true for children, because they can consume fairly small amounts of food, thereby possibly not introducing enough iron into the body, and also because they tend to be picky eaters. This was nicely demonstrated in a study of Szymlek-Gay performed in toddlers, where only 3.4% of children consumed the recommended amount of red meat dishes designed specifically for this population and ≈ 30% consumed half of the target amount. On the other hand, adherence to the consumption of fortified milk was above 80% [21].

Our study confirms that adherence to dietary advice is low; 4.7% of the children (four children) had modifications to increase iron intake introduced into their diet for the whole duration of the study, and only 23.5% (20) of participants managed to follow the altered dietary regimen for half the time during the study. These low proportions hinder statistically relevant estimations of the effect of altered dietary regimen on the results obtained. On the other hand, overall mean compliance to supplementation calculated from study diaries was 92%, not significantly differing between both study arms. This is important since good compliance to oral iron generally can be the decisive factor of the success of the intervention in individuals with ID and IDA, even more so than the type of iron included in the product [5].

To date, several applications of micronized and microencapsulated iron in the management of ID and IDA have been described, however, mostly as food fortification approaches in children as well as in adults [22,23,24,25,26,27]. Only recently, the results of studies on micronized, microencapsulated iron as stand-alone food supplements in the form of a powder [28], an oro-dispersible stick [29], or a tablet [30] in a population of generally healthy women with or without ID have been published. To our knowledge, the present study is the first one to report on the usefulness of micronized and microencapsulated iron as a stand-alone liquid food supplement in providing nutritional support in an ID pediatric population.

## Figures and Tables

**Table 1:** Participant demographics and study arm distribution.

	Placebo	>Your< Iron Syrup
N	21	64
Male	7 (33%)	36 (56%)
Female	14 (67%)	28 (44%)
Age (yrs.)	2.75 ± 1.50	3.20 ± 1.60
Height (cm)	93.3 ± 14.3	96.0 ± 16.0
Weight (kg)	13.9 ± 4.2	15.0 ± 4.2

Abbreviations: N, sample size. Age, height, and weight provided as mean ± SD.

**Table 2:** Comparison of participant hematological characteristics and iron status between baseline and the 4-week and 12-week follow-up.

Parameter	Arm	Baseline		FU at 4 Weeks		FU at 12 Weeks	
		Mean ± SD	*p*-Value	Mean ± SD	*p*-Value	Mean ± SD	*p*-Value
Ferritin level (µg/L)	P	14.0 ± 4.2 (21)	0.63	20.7 ± 11.8 (19)	0.14	19.6 ± 7.9 (21)	0.04 *
	YIS	14.5 ± 4.4 (64)		25.4 ± 12 (62)		24.5 ± 12.3 (64)	
Hemoglobin level (g/L)	P	118.0 ± 7.0 (21)	0.21	119 ± 6.9 (20)	0.13	117.0 ± 8.3 (19)	0.02
	YIS	121.0 ± 8.9 (64)		123.0 ± 10.2 (61)		122.0 ± 8.5 (62)	
Hematocrit (L/L)	P	0.36 ± 0.02 (21)	0.26	0.35 ± 0.02 (20)	0.06	0.35 ± 0.04 (19)	0.02
	YIS	0.36 ± 0.03 (64)		0.36 ± 0.02 (61)		0.36 ± 0.02 (62)	
MCV (fL)	P	77.1 ± 4.6 (21)	0.59	78.2 ± 4 (20)	0.11	78.2 ± 4.3 (19)	0.50
	YIS	76.4 ± 4.8 (64)		76.3 ± 4.7 (61)		77.4 ± 4.6 (62)	
MCH (pg)	P	25.8 ± 1.9 (21)	0.62	26.3 ± 1.4 (20)	0.13	26.3 ± 1.4 (20)	0.72
	YIS	25.5 ± 1.9 (64)		25.6 ± 1.9 (61)		25.9 ± 1.8 (62)	
MCHC (g/L)	P	333 ± 9 (21)	0.83	337 ± 7 (20)	0.99	334 ± 11 (19)	0.68
	YIS	333 ± 9 (64)		337 ± 15 (61)		335 ± 8 (62)	
Leukocyte count (10^9^)	P	8.3 ± 1.9 (20)	0.67	8 ± 2.2 (20)	0.06	8.6 ± 1.8 (19)	0.38
	YIS	8.5 ± 2.3 (64)		9.4 ± 3.1 (61)		10.5 ± 9 (62)	
Erythrocyte count (10^9^)	P	4.6 ± 0.3 (21)	0.12	4.5 ± 0.3 (20)	0.01	4.5 ± 0.4 (19)	0.03
	YIS	4.8 ± 0.4 (64)		4.8 ± 0.3 (61)		4.7 ± 0.4 (62)	
Thrombocyte count (10^9^)	P	346 ± 70 (21)	0.76	330 ± 67 (20)	0.98	359 ± 101 (19)	0.16
	YIS	339 ± 96 (64)		331 ± 81 (61)		322 ± 97 (62)	

Abbreviations: FU, follow-up; P, placebo; YIS, >Your< Iron Syrup; MCV, mean corpuscular volume; MCH, mean corpuscular hemoglobin; MCHC, mean corpuscular hemoglobin concentration. Numbers in brackets represent the number of analyzed individuals. * An independent-samples t-test where unequal variances are assumed is used because standard deviations differ between the study arms.

**Table 3:** Frequency and severity of reported adverse events.

Arm	AE Type	AE Severity and Causality												∑ (%)
		Mild				Moderate				Severe				
		Nn	Ps	Pb	Df	Nn	Ps	Pb	Df	Nn	Ps	Pb	Df	
**P**	**GIT**	5	7	/	/	1	2	1	/	/	/	/	/	16 (40)
	**Inf**	15	/	/	/	6	/	/	/	/	/	/	/	21 (48)
	**Skin**	1	/	/	/	/	/	/	/	/	/	/	/	1 (2)
	**Othr**	4	/	/	/	/	/	/	/	/	/	/	/	4 (10)
**∑ (%)**		32 (76)				10 (24)				/				42
**YIS**	**GIT**	28	7	4	/	2	2	3	/	1	1	/	/	48 (28)
	**Inf**	82	/	/	/	10	/	/	/	6	/	/	/	98 (57)
	**Skin**	5	1	/	/	/	/	/	/	/	/	/	/	6 (3)
	**Othr**	14	2	/	/	4	/	/	/	/	/	/	/	20 (12)
**∑ (%)**		143 (83)				21 (12)				8 (5)				172

Abbreviations: AE, adverse event; P, placebo; YIS, >Your< Iron Syrup; GIT, gastrointestinal adverse event; Inf, infectious disease; Othr, other; Nn, none; Ps, possible; Pb, probable; Df, definite.

**Table 4:** Compliance to syrup consumption as calculated from volumes of syrup in returned syrup bottles.

		100% Compliance		At Least 80% Compliance	
		Yes	No	Yes	No
Placebo arm	N	18	3	21	0
	%	86	14	100	0
YIS arm	N	51	13	61	3
	%	80	20	95	5
Total	N	69	16	82	3
	%	80	20	97	3
p value		0.54		0.31	

Abbreviations: YIS, >Your< Iron Syrup. Compared with Fisher’s exact test.

## Data Availability

The data that support the findings of this study are available from the corresponding author, J.J., upon reasonable request.

## References

[1] Pasricha S.R., Hayes E., Kalumba K., Biggs B.A. (2013). Effect of daily iron supplementation on health in children aged 4-23 months: A systematic review and meta-analysis of randomised controlled trials. Lancet Glob. Health.

[2] McDermid J.M., Lönnerdal B. (2012). Iron. Adv. Nutr..

[3] Georgieff M.K. (2011). Long-term brain and behavioral consequences of early iron deficiency. Nutr. Rev..

[4] Domellöf M., Braegger C., Campoy C., Colomb V., Decsi T., Fewtrell M., Hojsak I., Mihatsch W., Molgaard C., Shamir R. (2014). Iron requirements of infants and toddlers. J. Pediatric Gastroenterol. Nutr..

[5] Camaschella C. (2015). Iron-deficiency anemia. N. Engl. J. Med..

[6] WHO (2017). Nutritional Anaemias: Tools for Effective Prevention and Control.

[7] WHO (2011). Guideline: Intermittent Iron Supplementation in Preschool and School-Age Children.

[8] WHO (2020). Guideline on Use of Ferritin Concentrations To Assess Iron Status in Individuals and Populations.

[9] WHO (2015). The Global Prevalence of Anaemia in 2011.

[10] Clénin G.E. (2017). The treatment of iron deficiency without anaemia (in otherwise healthy persons). Swiss Med. Wkly..

[11] Thompson J., Biggs B.A., Pasricha S.R. (2013). Effects of daily iron supplementation in 2- to 5-year-old children: Systematic review and meta-analysis. Pediatrics.

[12] Ullrich C., Wu A., Armsby C., Rieber S., Wingerter S., Brugnara C., Deficiency R.O.N., The I.S., Com M. (2005). Using Reticulocyte Hemoglobin Content. Am. J. Hematol..

[13] Soppi E. (2019). Iron Deficiency Without Anemia–Common, Important, Neglected. Clin. Case Rep. Rev..

[14] Pachuta Węgier L., Kubiak M., Liebert A., Clavel T., Montagne A., Stennevin A., Roye S., Boudribila A. (2020). Ferrous sulfate oral solution in young children with iron deficiency anemia: An open-label trial of efficacy, safety, and acceptability. Pediatric Int..

[15] Hawamdeh H.M., Rawashdeh M., Aughsteen A.A. (2013). Comparison between once weekly, twice weekly, and daily oral iron therapy in Jordanian children suffering from iron deficiency anemia. Matern. Child Health J..

[16] Faqih A.M., Kakish S.B., Izzat M. (2006). Effectiveness of intermittent iron treatment of two- to six-year-old Jordanian children with iron-deficiency anemia. Food Nutr. Bull..

[17] Arora S., Kapoor R.K. (2012). Iron metabolism in humans: An Overview. InTech.

[18] Choi H.S., Song S.H., Lee J.H., Kim H.J., Yang H.R. (2012). Serum hepcidin levels and iron parameters in children with iron deficiency. Korean J. Hematol..

[19] Suganya V., Anuradha V. (2017). Microencapsulation and Nanoencapsulation: A Review. Int. J. Pharm. Clin. Res..

[20] Finlayson-Trick E.C., Fischer J.A., Goldfarb D.M., Karakochuk C.D. (2020). The Effects of Iron Supplementation and Fortification on the Gut Microbiota: A Review. Gastrointest. Disord..

[21] Szymlek-Gay E.A., Ferguson E.L., Heath A.L.M., Gray A.R., Gibson R.S. (2009). Food-based strategies improve iron status in toddlers: A randomized controlled trial. Am. J. Clin. Nutr..

[22] Fidler M.C., Walczyk T., Davidsson L., Zeder C., Sakaguchi N., Juneja L.R., Hurrell R.F. (2004). A micronised, dispersible ferric pyrophosphate with high relative bioavailability in man. Br. J. Nutr..

[23] Waller A.W., Andrade J.E., Mejia L.A. (2020). Performance factors influencing efficacy and effectiveness of iron fortification programs of condiments for improving anemia prevalence and iron status in populations: A systematic review. Nutrients.

[24] Blanco-Rojo R., Pérez-Granados A.M., Toxqui L., González-Vizcayno C., Delgado M.A., Vaquero M.P. (2011). Efficacy of a microencapsulated iron pyrophosphate-fortified fruit juice: A randomised, double-blind, placebo-controlled study in Spanish iron-deficient women. Br. J. Nutr..

[25] Navas-Carretero S., Pérez-Granados A.M., Sarriá B., Vaquero M.P. (2009). Iron absorption from meat pate fortified with ferric pyrophosphate in iron-deficient women. Nutrition.

[26] Moretti D., Zimmermann M.B., Muthayya S., Thankachan P., Lee T.C., Kurpad A.V., Hurrell R.F. (2006). Extruded rice fortified with micronized ground ferric pyrophosphate reduces iron deficiency in Indian schoolchildren: A double-blind randomized controlled trial1-3. Am. J. Clin. Nutr..

[27] Roe M.A., Collings R., Hoogewerff J., Fairweather-Tait S.J. (2009). Relative bioavailability of micronized, dispersible ferric pyrophosphate added to an apple juice drink. Eur. J. Nutr..

[28] Hussain U., Zia K., Iqbal R., Saeed M., Ashraf N. (2019). Efficacy of a Novel Food Supplement (Ferfer^®^) Containing Microencapsulated Iron in Liposomal Form in Female Iron Deficiency Anemia. Cureus.

[29] Ple A., Ple C., Rosoga N., Nedelcu S. (2015). Efficacy and tolerability of a novel food supplement (Turbofer^®^) containing microencapsulated iron in liposomal form, in female iron deficiency anemia. J. Nutr. Int. Med..

[30] Parisi F., Berti C., Mandò C., Martinelli A., Mazzali C., Cetin I. (2017). Effects of different regimens of iron prophylaxis on maternal iron status and pregnancy outcome: A randomized control trial. J. Matern. Neonatal Med..

